# Zyxin Inhibits the Proliferation, Migration, and Invasion of Osteosarcoma via Rap1-Mediated Inhibition of the MEK/ERK Signaling Pathway

**DOI:** 10.3390/biomedicines11082314

**Published:** 2023-08-21

**Authors:** Zhun Wei, Kezhou Xia, Bin Zhou, Di Zheng, Weichun Guo

**Affiliations:** 1 Department of Orthopedics, Renmin Hospital of Wuhan University, Wuhan 430060, China; 2 Department of Orthopedics, Ezhou Central Hospital, Ezhou 436000, China

**Keywords:** ZYX, osteosarcoma, proliferation, MEK/ERK pathway, Rap1

## Abstract

Zyxin (ZYX) is an actin-interacting protein with unknown biological functions in patients with osteosarcoma. This research sought to understand how ZYX affects the biological behavior of osteosarcoma cells and to identify the associated mechanism. Firstly, ZYX expression was decreased in osteosarcoma, and its higher expression indicated better outcomes in patients with osteosarcoma. ZYX overexpression significantly inhibited the proliferation, migration, and invasion of osteosarcoma cells, whereas ZYX silencing resulted in the opposite trend. Subsequently, we found that the Rap1 signaling pathway was significantly correlated with ZYX expression as reported in The Cancer Genome Atlas’s database using bioinformatic analysis. Moreover, we found that ZYX overexpression regulated the Rap1/MEK/ERK axis, and osteosarcoma cell growth, migration, and invasion were consequently restrained. Additionally, by administering tumor cells subcutaneously to nude mice, a mouse model of transplanted tumors was created. Compared to the control group, the ZYX overexpression group’s tumors were lighter and smaller, and the ZYX/Rap1 axis was activated in the ZYX overexpression group. Taken together, our results suggest that ZYX inhibits osteosarcoma cell proliferation, migration, and invasion by regulating the Rap1/MEK/ERK signaling pathway. ZYX might be crucial in the clinical management of osteosarcoma and is a promising novel therapeutic target in patients with this disease.

## 1. Introduction

Osteosarcoma is the most common malignant bone tumor in individuals aged between 5 and 20 years [1,2]. It often occurs in the distal femur and proximal tibia, and the main symptoms are local swelling and pain [3]. Patients with advanced osteosarcoma have poor prognoses and are prone to lung metastasis, despite substantial advancements in osteosarcoma diagnosis and treatment [4,5,6]. Currently, the main treatment methods are neoadjuvant chemotherapy combined with surgical excision, including preoperative chemotherapy, intraoperative radical excision, and postoperative chemotherapy [7]. Although this advanced treatment improves the 5-year survival rate from 20% to 70% in patients with osteosarcoma without metastasis, the rate remains at 20% in those with metastasis [7,8]. Uncontrolled migration and invasion are the main reasons for the high malignancy of tumors and are accompanied by complex molecular biological regulation mechanisms [9,10]. Therefore, determining the biological mechanism underpinning the migration and invasion of osteosarcoma is crucial for clinically treating patients with osteosarcoma and improving their prognoses; however, research on this is lacking [11]. Therefore, novel therapeutic targets for the clinical treatment of osteosarcoma must be identified urgently.

Zyxin (ZYX) is an actin-interacting protein that contributes to the centripetal movement of cell–cell connections and is consistent with the systolic actomyosin network [12,13]. It is localized to adhesion plaques and junctions and is involved in regulating cell adhesion, migration, and actin morphological remodeling [14,15,16]. Numerous studies have been conducted on ZYX’s role in cancer. Zhong et al. first demonstrated that decreased ZYX expression may inhibit the proliferation and metastasis of colorectal cancer cells via the FA pathway [16]. ZYX acts as an oncogenic gene in glioblastoma multiforme cells, and higher ZYX expression promotes the metastasis of glioblastoma multiforme cells by regulating the transcription of STMN1 [14]. Another study reported decreased ZYX expression, disrupted cell adhesion, and enhanced cell invasion and migration in non-small-cell lung cancer patients [17]. These studies suggest that ZYX has different or even opposite effects on various tumors. Nevertheless, it is still unknown how ZYX affects patients with osteosarcoma, and the underlying mechanism warrants further study.

In this study, we found that in patients with osteosarcoma, ZYX was downregulated, and lower expression levels of ZYX predicted a poor prognosis. The osteosarcoma was prevented from growing, migrating, and invading both in vitro and in vivo when ZYX was overexpressed. Conversely, ZYX knockdown had the opposite effect. Mechanistically, ZYX inhibited osteosarcoma progression by binding to Rap1, thereby inhibiting the MEK/ERK signaling pathway. Our study revealed a specific role of the ZYX/Rap1 axis in the progression of osteosarcoma. ZYX is a promising novel therapeutic target for patients with osteosarcoma and may be crucial in the clinical therapy of this illness.

## 2. Materials and Methods

### 2.1. Sample Collection

Samples of osteosarcoma and nearby healthy regions were obtained from Renmin Hospital of Wuhan University (Wuhan, China) between June 2021 and March 2023 (Table 1). After being surgically removed, all tissues were placed in liquid nitrogen storage before being used for further research. The use of patient tissue samples was approved by the Ethics Committee of Renmin Hospital of Wuhan University.

### 2.2. Bioinformatic Analysis

To examine the differential expression of ZYX in various tumors, the Tumor Immune Estimation Resource (TIMER) database (https://cistrome.shinyapps.io/timer/, accessed on 1 June 2023) was used. The Cancer Genome Atlas (TCGA)’s database was also used to download transcriptome sequencing data and clinical information on osteosarcoma and to then evaluate the prognostic efficacy of ZYX in patients with osteosarcoma.

### 2.3. Cell Culture and Transfection

We obtained 143B and U2OS osteosarcoma cell lines from ProCell Technology (Wuhan, China). Human osteoblasts (hFOB1.19) were purchased from the China Center for Type Culture Collection (Wuhan, China). We cultured the 143B and hFOB1.19 cells in an RPMI 1640 medium (Invitrogen, Carlsbad, CA, USA) and the U2OS cells in Dulbecco’s Modified Eagle Medium (Invitrogen). Additionally, 10% fetal bovine serum (from Tian Hang, Huzhou, China) and 1% antibodies (100 mg/mL streptomycin and 100 U/mL penicillin) were added to all media as a supplement. Osteosarcoma cells were grown in an incubator set to a constant 37 °C and 5% CO_2_ atmosphere, while the incubation temperature of hFOB1.19 was 34 °C. For lentivirus infection, we constructed stably transfected cells using lentivirus constructed by OBiO Technology (Shanghai, China). The constructs in this study were as follows: LV-Control: pcSLenti-EF1-EGFP-P2A-Puro-CMV-MCS-3×flag-WPRE; LV-ZYX: pcSLenti-EF1-EGFP-P2A-Puro-CMV-ZYX-3×flag-WPRE; shZYX: psLenti-EF1a-EGFP-P2A-Puro-U6-shZYX-WPRE; shRap1: psLenti-EF1a-EGFP-P2A-Puro-U6-shRap1-WPRE; shNC: psLenti-EF1a-EGFP-P2A-Puro-U6-shNC-WPRE. We used 5 μg/mL polybrene to promote transfection efficiency. Furthermore, stably transfected cells were examined with puromycin at a concentration of 5 μg/mL. The effectiveness of transfection was then assessed using a Western blot analysis and a quantitative reverse transcription polymerase chain reaction (qRT-PCR) assay.

### 2.4. Cell Counting Kit-8 Assay

The Cell Counting Kit (CCK)-8 assay was used to detect whether cells were proliferating. Trypsin was used to initially break down the cells (Servicebio Technology, Wuhan, China), after which they were resuspended in media. Then, 1000 cells were plated onto 96-well plates. Phosphate-buffered saline (PBS) was used to wash the cells after the growth media was removed. On days 1, 2, 3, 4, and 5, we applied 10 μL of the CCK-8 reagent (Servicebio Technology) to each well. Cell viability was assessed using a microplate reader (Bio-Rad Laboratories Inc., Hercules, CA, USA) to measure absorbance at 450 nm after 1 h of incubation in complete darkness.

### 2.5. Wound-Healing Assay 

First, 5 × 10^5^ cells were plated in a six-well plate and cultured for 48 h. Using a 1 mL pipette tip, a light straight line was softly drawn over the cell surface when cell density reached 80%. Second, the cells were incubated with a serum-free medium, and the scratched areas were observed after 36 h of incubation. The ability of the cells to migrate was quantified by calculating the scratch area as follows: [1 − (empty area 36 h/empty area 0 h)] × 100%.

### 2.6. Transwell Invasion Assay

Transwell chambers (Corning Inc., Corning, NY, USA) and Matrigel (BD Biosciences, Franklin Lakes, NJ, USA) were used for the Transwell invasion assays. The upper chamber was first filled with 80 μL of Matrigel. Thereafter, a 500 μL medium containing 20% serum was added to the lower chamber. After the Matrigel solidified in the upper chamber, 1 × 10^5^ cells cultured in 10% serum were added to each upper chamber. The upper chamber was treated with 4% paraformaldehyde for 15 min after 48 h, followed by three PBS washes and 15 min of crystal violet staining. Finally, a microscope with an inverted viewing position (Olympus, Tokyo, Japan) was used to examine the stained top chamber.

### 2.7. Western Blot Analysis

Total protein was extracted from the 143B, U2OS, MG63, HOS, and hFOB1.19 cell lines using a RIPA lysis buffer (Servicebio Technology). Concentrations of the extracted proteins were determined using a bicinchoninic acid kit (Beyotime Biotechnology, Beijing, China). The proteins were subjected to 10% sodium dodecyl sulfate-polyacrylamide gel electrophoresis at 120 V for 1 h. The bands were then blocked with 5% skim milk for 1 h at room temperature after the proteins were transferred to a polyvinylidene fluoride membrane with a 200 mA current for 100 min. Next, the bands were subjected to three Tris-buffered saline with 0.1% Tween (TBST) washes before being incubated with primary antibodies (Table 2) for an entire night at 4 °C. The bands were incubated with a secondary antibody for 1 h at room temperature the following day after being washed three times with TBST. Finally, an electrochemiluminescence detection kit (Biosharp Biotechnology, Hefei, China) was used to visualize the blots.

### 2.8. Quantitative Real-Time Polymerase Chain Reaction (qRT-PCR) 

Total RNA was extracted from the 143B, U2OS, MG63, HOS, and hFOB1.19 lines using TRIzol reagent (Servicebio, Wuhan). cDNA was synthesized by employing SweScript All-in-One First-Strand cDNA and Synthesis SuperMix for qPCR (gDNA Remover) (Servicebio, Wuhan). cDNA synthesis system (20 μL) contained 4 μL of SweScript, 1 μL of gDNA Remover, 10 μL of total RNA, and 5 μL of RNase free water. cDNA was synthesized according to the manufacturer’s instructions: treatment at 25 °C for 5 min, 42 °C for 30 min, and finally 85 °C for 5 s. qRT-PCR was conducted using SYBR Green qPCR Master Mix (Servicebio, Wuhan). The PCR system (20 μL) contained 10 μL of SYBR, 1 μL 10 μM of forward and 1 μL 10 μM of reverse primers, 2 μL of cDNA, and 6 μL of nuclease-free water. According to the manufacturer’s instructions, qRT-PCR was performed using the following thermal cycle conditions: predenaturation at 95 °C for 30 s; 40 cycles performed at 95 °C for 15 s and 60 °C for 30 s; finally, 72 °C for 5 min. Table 3 lists the sequences of the two primers used. Relative gene expression was calculated using the ΔΔCT method.

### 2.9. Xenograft Tumor Model

The Ethics Committee of Renmin Hospital of Wuhan University approved all animal trials. The 4–5-week-old nude mice were acquired from Wuhan Shulaibao Biotechnology Co., Ltd. (Wuhan, China) and nurtured in the Renmin Hospital of Wuhan University’s Animal Experimental Center. All mice were kept in a specific pathogen-free (SPF) environment and given access to enough food and water. All mice were evenly divided into two groups (LV-Control and LV-ZYX) with six mice in each group. A total of 5 ×10^6^ stably transfected 143B osteosarcoma cells (LV-Control and LV-ZYX) were administered subcutaneously on the forelimb axillary fossa of the animals in each group. Subsequently, tumor volume and weight were monitored every 7 days, and all mice were sacrificed 4 weeks after injection. The tumors were preserved in 4% paraformaldehyde or liquid nitrogen for subsequent experiments.

### 2.10. Immunohistochemistry Staining

Rap1, Ki-67, and p-ERK were detected using immunohistochemistry (IHC), as previously described [18]. Briefly, 4 μm of paraffin-embedded tissues were dewaxed, and the antigens were obtained at 95 °C (10 min) using a citric acid buffer followed by treatment with 0.3% H_2_O_2_ (10 min). The sections were then treated with primary antibodies at 4 °C overnight after being blocked with 5% BSA for 1 h. The primary antibodies used were anti-Rap1 (1:200; CST, USA), anti–Ki-67 (1:200; CST, USA), and anti–p-ERK (1:200; CST, USA). The next day, the sections were incubated with a secondary antibody (Servicebio Technology) at 37 °C and stained using diaminobenzidine. Finally, the sections were stained with hematoxylin and eosin and observed under a light microscope (Olympus).

### 2.11. Statistical Analysis

GraphPad Prism, version 8.0 (GraphPad Software, San Diego, CA, USA) and R, version 4.2.2 (The R Foundation for Statistical Computing, Vienna, Austria) were used to perform statistical analysis. All our experiments were repeated three times. The data from the two groups were analyzed using one-way analysis of variance, and the difference between the two groups was compared using Student’s t-test. Chi-square test was used to determine the correlation between ZYX expression and clinicopathological parameters. Unless specified otherwise, all results in our study are presented as mean ± standard deviation. A *p*-value < 0.05 was considered statistically significant. *** indicates *p* < 0.001, ** indicates *p* < 0.01, and * indicates *p* < 0.05.

## 3. Results

### 3.1. ZYX Was Downregulated in Osteosarcoma Tissues, and Its Low Expression Predicted Poor Prognoses in Patients with Osteosarcoma

**Figure 1:**
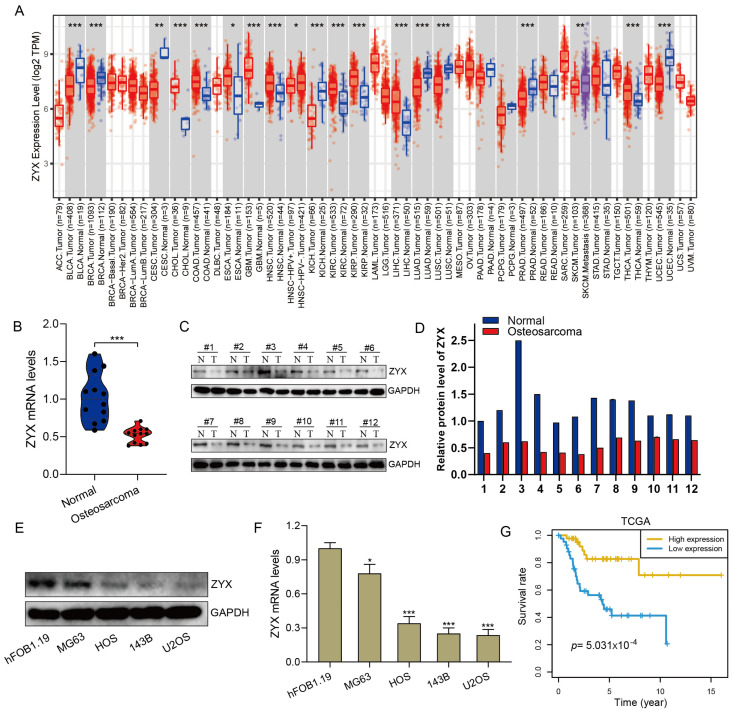
ZYX expression is significantly reduced in osteosarcoma cells and tissues and predicts a poor prognosis in patients with osteosarcoma. (**A**) The expression of ZYX in various tumors was reported on the TIMER website based on TCGA’s database. (**B**) The expression of ZYX in normal tissues and osteosarcoma tissues was detected by qRT-PCR. (**C**,**D**) Western blot and quantitative analysis of the expression level of ZYX in osteosarcoma tissues and normal tissues. (**E**,**F**) Western blot and quantitative analysis of the expression level of ZYX in osteosarcoma cells (MG63, HOS, 143B, and U2OS) and normal osteoblast cells (hFOB1.19). GAPDH is used as an internal reference. (**G**) Kaplan–Meier survival analysis in TCGA’s database suggested that a higher expression level of ZYX was associated with a better prognosis. All data are from three independent experiments and are shown as mean ± SD. * *p <* 0.05; ** *p <* 0.01; *** *p <* 0.001.

Using the TIMER database, we analyzed the expression levels of ZYX in various cancers. As shown in Figure 1A, ZYX was expressed at higher levels in cholangiocarcinoma (CHOL), colon adenocarcinoma (COAD), esophageal carcinoma (ESCA), glioblastoma (GBM), head and neck squamous cell carcinoma (HNSC), kidney renal clear cell carcinoma (KIRC), kidney renal papillary cell carcinoma (KIRP), liver hepatocellular carcinoma (LIHC), and thyroid carcinoma (THCA) than in normal tissues, whereas it was expressed at lower levels in bladder urothelial carcinoma (BLCA), breast invasive carcinoma (BRCA), cervical squamous cell carcinoma and endocervical adenocarcinoma (CESC), kidney chromophobe (KICH), lung adenocarcinoma (LUAD), lung squamous cell carcinoma (LUSC), prostate adenocarcinoma (PRAD), and uterine corpus endometrial carcinoma (UCEC). The role of ZYX in osteosarcoma tissue, however, is poorly understood. We evaluated ZYX expression levels in osteosarcoma cell lines as well as tissue samples. ZYX mRNA and protein seemed to be expressed at lower levels in osteosarcoma tissues than in healthy adjacent tissues, according to qRT-PCR and Western blot assays. (Figure 1B–D). Moreover, ZYX protein levels were significantly lower in osteosarcoma cells (MG63, HOS, 143B, and U2OS) than in osteoblasts (hFOB1.19) (Figure 1E,F). Higher expression levels of ZYX were associated with a better prognosis in patients with osteosarcoma, according to a Kaplan–Meier survival analysis of the data from TCGA’s database. (Figure 1G).

### 3.2. Upregulation of ZYX Restrains the Proliferation, Migration, and Invasion of Osteosarcoma Cells

**Figure 2:**
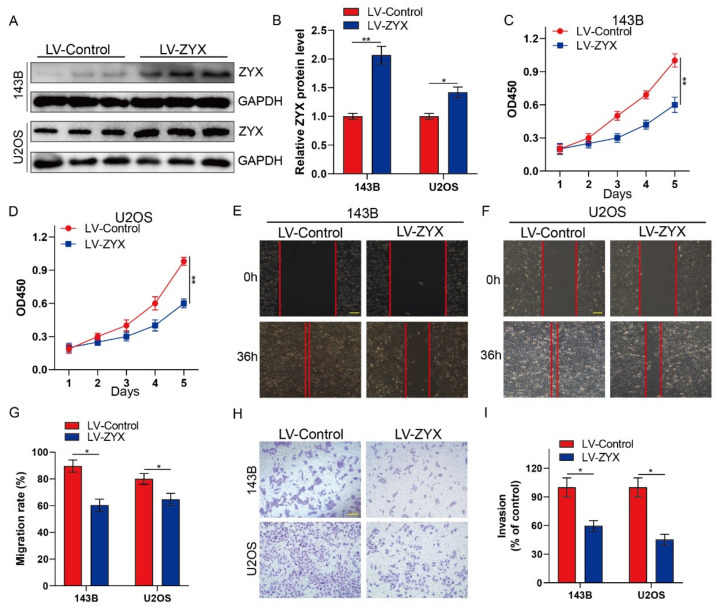
Overexpression of ZYX restrains the proliferation, migration, and invasion of osteosarcoma cells. (**A**,**B**) Western blot and quantitative analysis of ZYX in 143B and U2OS cells after overexpressing ZYX using lentivirus. GAPDH is used as an internal reference. (**C**,**D**) CCK-8 assay was performed in 143B and U2OS cells after ZYX overexpression. (**E**–**G**) Wound-healing assay and its quantitative analysis after overexpressing ZYX. Scale bar: 200 μm. (**H**,**I**) Transwell invasion assay in 143B and U2OS cells after ZYX overexpression and its quantitative analysis. Scale bar: 400 μm. All data are from three independent experiments and are shown as mean ± SD. * *p <* 0.05; ** *p <* 0.01.

A recombinant lentiviral vector was constructed to increase ZYX expression in order to investigate the function of ZYX in osteosarcoma cells. Western blot analysis confirmed that ZYX was stably overexpressed in 143B and U2OS cells (Figure 2A,B). The CCK-8 assay revealed that the ability of 143B and U2OS osteosarcoma cells to proliferate was markedly reduced when ZYX was overexpressed (Figure 2C,D). The wound-healing assay revealed that the LV-ZYX group’s healing area was significantly larger than that of the LV-Control group, indicating that the overexpression of ZYX prevents osteosarcoma cells from migrating (Figure 2E–G). Moreover, the Transwell invasion assay showed that ZYX overexpression reduced cellular invasion in osteosarcoma cells (Figure 2H,I). Collectively, these results revealed that ZYX upregulation inhibited the proliferation, migration, and invasion of osteosarcoma cells.

### 3.3. Silencing ZYX Promotes Osteosarcoma Cell Proliferation, Migration, and Invasion

**Figure 3:**
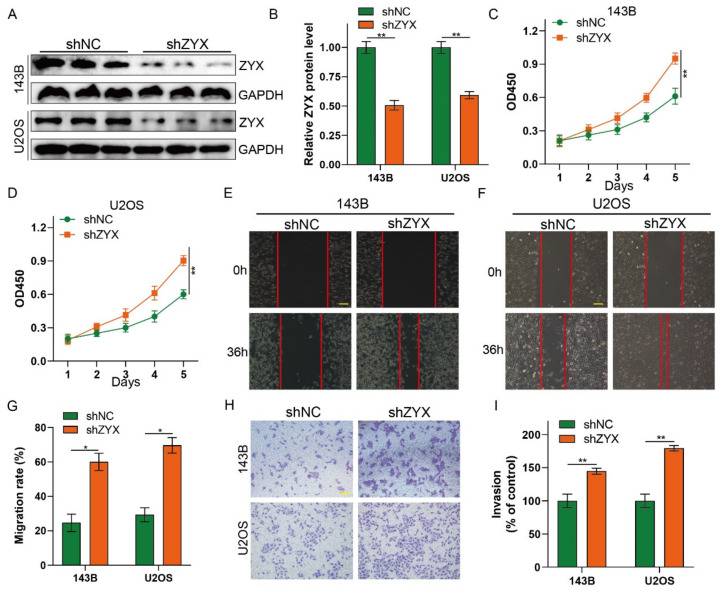
Silencing ZYX promotes the proliferation, migration, and invasion of osteosarcoma cells. (**A**,**B**) Western blot and quantitative analysis of ZYX in 143B and U2OS cells after transfection with shZYX. GAPDH is used as an internal reference. (**C**,**D**) CCK-8 assay was performed in 143B and U2OS cells after ZYX knockdown. (**E**–**G**) Wound-healing assay and its quantitative analysis after silencing ZYX. Scale bar: 200 μm. (**H**,**I**) Transwell invasion assay and its quantitative analysis in 143B and U2OS cells after ZYX knockdown. Scale bar: 400 μm. All data are from three independent experiments and are shown as mean ± SD. * *p <* 0.05; ** *p <* 0.01.

Since ZYX acts as a tumor inhibitor in osteosarcoma proliferation, we speculated whether silencing ZYX may have the opposite effect. To confirm this, ZYX-shRNAs and shNC cells were transfected into 143B and U2OS cell lines. Knockdown efficiency was confirmed by Western blot analysis (Figure 3A,B). Osteosarcoma cell proliferation was dramatically increased when ZYX was silenced, according to the CCK-8 assay (Figure 3C,D). Additionally, the Transwell invasion assays and wound-healing experiments revealed that ZYX downregulation facilitated osteosarcoma cell invasion and migration (Figure 3E–I). In summary, our results showed that silencing ZYX promotes the aggressiveness of osteosarcoma.

### 3.4. Differential Gene Expression, Kyoto Encyclopedia of Genes and Genomes Enrichment Analysis, and Molecular Docking

**Figure 4:**
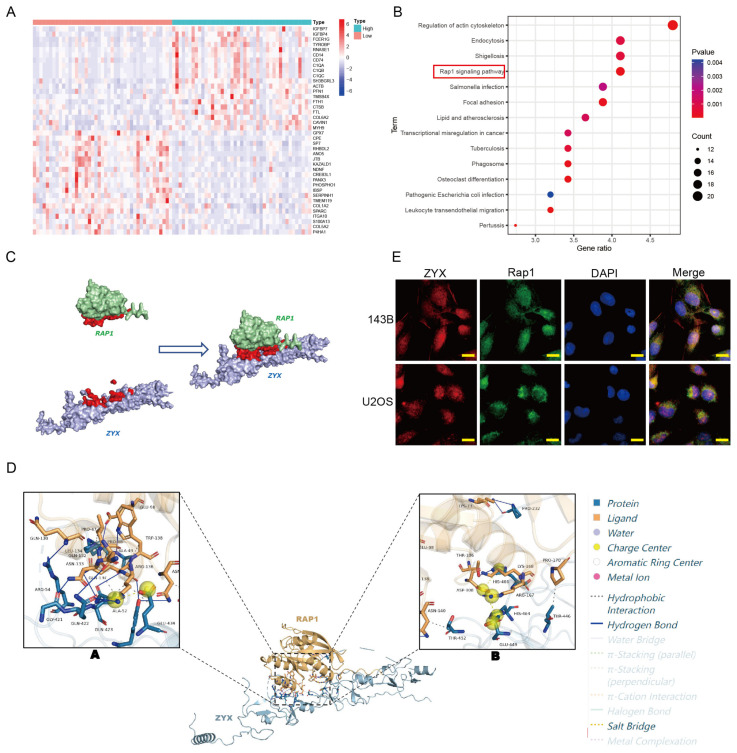
Differential gene expression, KEGG enrichment analysis, and molecular docking. (**A**) Heatmap exhibits the differentially expressed genes in TCGA’s database. (**B**) KEGG enrichment analysis of the differentially expressed genes revealed that Rap1 signaling pathway was significantly enriched. (**C**,**D**) Molecular docking suggested that ZYX and Rap1 can interact with each other. (**E**) Co-localization of ZYX and Rap1 in 143B and U2OS cells. Scale bar: 20 μm. All data are from three independent experiments and are shown as mean ± SD.

To further investigate the molecular mechanism of the ZYX-mediated inhibition of the proliferation, migration, and invasion of osteosarcoma cells, we analyzed the differential genes between samples with highly and poorly expressed ZYX in TCGA’s database (Figure 4A). The analysis of the Kyoto Encyclopedia of Genes and Genomes (KEGG) enrichment data demonstrated a substantial correlation between the Rap1 signaling pathway and ZYX expression (Figure 4B). To further explore the relationship between ZYX and Rap1, molecular docking was performed, and the results showed that ZYX interacted with Rap1 (Figure 4C,D). Cellular immunofluorescence experiments also confirmed the co-localization of ZYX and Rap1 in 143B and U2OS cells (Figure 4E).

### 3.5. Overexpression of ZYX Regulates the Rap1/MEK/ERK Axis in Osteosarcoma

**Figure 5:**
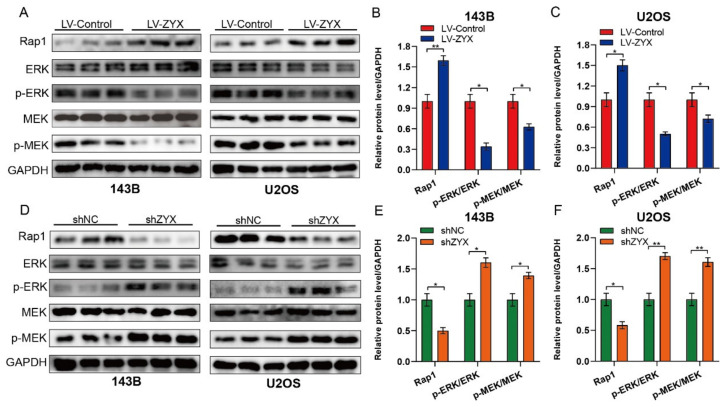
ZYX regulates Rap1/MEK/ERK axis in osteosarcoma cells. (**A**–**C**) Western blot and quantitative analysis of protein level of Rap1, ERK, p-ERK, MEK, and p-MEK in 143B and U2OS cells after ZYX overexpression. (**D**–**F**) Western blot and quantitative analysis of protein level of Rap1, ERK, p-ERK, MEK, p-MEK in 143B and U2OS cells with ZYX knockdown. GAPDH is used as an internal reference. All data are from three independent experiments and are shown as mean ± SD. * *p <* 0.05; ** *p <* 0.01.

Since Rap1 signaling is enhanced following ZYX overexpression, and the MEK/ERK signaling pathway has been proven to be directly downstream of Rap1 [19,20,21], we further explored the effect of ZYX on the Rap1/MEK/ERK signaling pathway. Rap1 protein expression was elevated as a result of ZYX overexpression, but ERK and MEK phosphorylation was decreased (Figure 5A–C). In contrast, ZYX silencing dramatically reduced Rap1 protein expression while increasing ERK and MEK phosphorylation (Figure 5D,F). In summary, these findings imply that ZYX is related to the Rap1/MEK/ERK signaling pathway in osteosarcoma.

### 3.6. ZYX Inhibits Osteosarcoma Cell Proliferation, Migration, and Invasion by Regulating the Rap1/MEK/ERK Signaling Pathway

**Figure 6:**
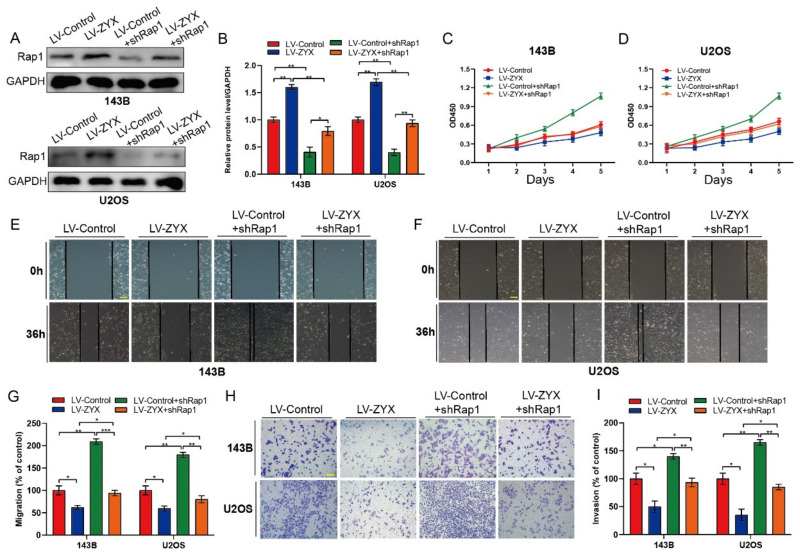
ZYX restrains osteosarcoma cell proliferation, migration, and invasion by regulating the Rap1/MEK/ERK signaling pathway. (**A**,**B**) Western blot and quantitative analysis confirmed the shRNA efficiency in 143B and U2OS cells. GAPDH is used as an internal reference. (**C**,**D**) CCK-8 assay suggested that silencing Rap1 attenuated the inhibition of ZYX overexpression on the proliferation of osteosarcoma cells. (**E**–**G**) Wound-healing assay and its quantitative analysis revealed that knockdown of Rap1 can significantly attenuate the inhibitory effect of ZYX overexpression on the migration ability of osteosarcoma cells. Scale bar: 200 μm. (**H**,**I**) Transwell invasion assay and its quantitative analysis showed that silencing Rap1 impaired the inhibitory effect of ZYX overexpression on the invasion ability of osteosarcoma cells. Scale bar: 400 μm. All data are from three independent experiments and are shown as mean ± SD. * *p <* 0.05; ** *p <* 0.01; *** *p <* 0.001.

To more clearly define the mechanism by which ZYX prevents osteosarcoma cells from proliferating, migrating, and invading, Rap1-shRNAs were transfected into osteosarcoma cell lines. Western blot analysis confirmed shRNA efficiency in 143B and U2OS cells (Figure 6A,B). The CCK-8 experiment revealed that the inhibition of osteosarcoma cell proliferation by ZYX overexpression may be compromised by Rap1 downregulation. (Figure 6C,D). Similarly, in the wound-healing assay, Rap1 knockdown dramatically reduced the inhibitory effect of ZYX overexpression on the ability of osteosarcoma cells to migrate. (Figure 6E–G). In comparison to the LV-ZYX group, osteosarcoma cells had a much higher ability to invade in the Transwell invasion assay after knocking down Rap1 (Figure 6H,I). These findings demonstrated that Rap1 downregulation dramatically reduces ZYX’s inhibitory effect on osteosarcoma cell proliferation, migration, and invasion.

### 3.7. ZYX Inhibits the Progression of Osteosarcoma in Vivo by Regulating the Rap1/MEK/ERK Signaling Pathway

**Figure 7:**
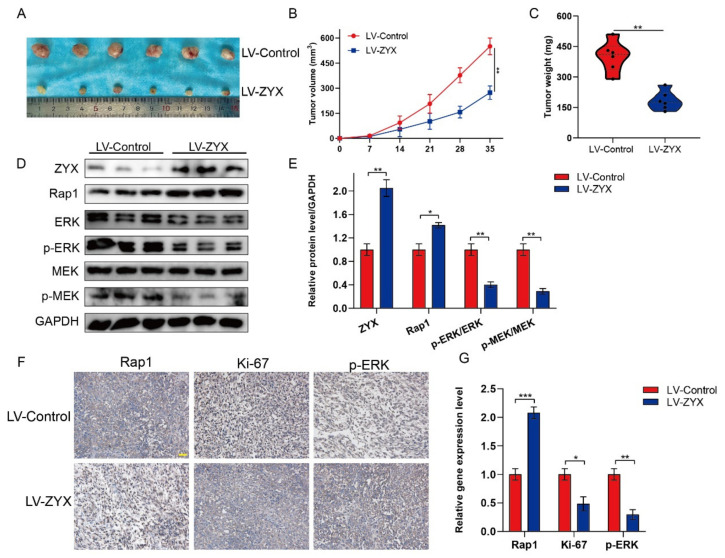
ZYX inhibits the progression of osteosarcoma in vivo via the Rap1/MEK/ERK axis. (**A**) Presentation of the transplanted tumor excised from nude mice in LV-Control and LV-ZYX groups. (**B**) The growth curves of tumor xenografts in nude mice in LV-Control and LV-ZYX groups. (**C**) Comparison of tumor weight of tumors excised from nude mice in LV-Control and LV-ZYX groups. (**D**,**E**) Western blot and quantitative analysis of Rap1, MEK, p-MEK, ERK, and p-ERK in tumors excised from nude mice in LV-Control and LV-ZYX groups. GAPDH is used as an internal reference. (**F**,**G**) Immunohistochemical analysis of Rap1, Ki-67, and p-ERK in tumors excised from two groups. Scale bar: 200 μm. All data are from three independent experiments and are shown as mean ± SD. * *p <* 0.05; ** *p <* 0.01; *** *p <* 0.001.

Our results showed that ZYX inhibited osteosarcoma development in vitro. We also explored the role of ZYX in the regulation of osteosarcoma in vivo. Accordingly, nude mice were used to create a xenograft model. Nude mice were subcutaneously injected with LV-Control and LV-ZYX 143B osteosarcoma cells (six mice per group), and the tumor volume and weight were assessed weekly. As shown in Figure 7A–C, mice in the LV-ZYX group were significantly smaller and weighed less than those in the LV-Control group. Moreover, the Western blot analysis of markers related to the Rap1/MEK/ERK signaling pathway in subcutaneous tumors indicated that Rap1 expression was considerably lower in the LV-ZYX group than in the LV-Control group, and Rap1 downregulation resulted in lower levels of p-ERK and p-MEK expressions (Figure 7D,E). IHC staining suggested that ZYX overexpression inhibited osteosarcoma proliferation and regulated the Rap1/MEK/ERK signaling pathway (Figure 7F,G). Therefore, we can deduce that the overexpression of ZYX significantly inhibits osteosarcoma progression, which is mediated by the regulation of the Rap1/MEK/ERK signaling pathway.

## 4. Discussion

As a key molecule in the regulation of focal adhesion, Legerstee et al. found in the U2OS cell line that ZYX mediates cell adhesion and migration and is critical for many pathophysiological processes [22]. ZYX plays an important role in the control of mitotic progression by interacting with h-warts/LATS1 to form a regulatory complex on the mitotic apparatus [23]. In addition, Tang et al. revealed that ZYX has high accuracy in predicting the prognosis of patients with osteosarcoma and is involved in the immune-related regulation of osteosarcoma [24]. Meanwhile, previous research has demonstrated that ZYX substantially increases the malignancy of glioblastoma and colon cancer while suppressing the growth of non-small-cell lung cancer in patients [14,17,25]. In the present study, using large independent human databases, we found that ZYX is differentially expressed in various human tumors and normal tissues. Clinically, osteosarcoma tissues exhibit considerably lower ZYX expression levels than normal tissues, and low ZYX expression in humans predicts a poor prognosis. Moreover, ZYX overexpression prevented osteosarcoma cells from proliferating, migrating, and invading, whereas ZYX silencing showed the opposite effect, further supporting the idea that ZYX acts as a tumor suppressor in patients with osteosarcoma.

We analyzed the differential genes between samples with highly and poorly expressed ZYX in TCGA’s database, and KEGG enrichment analysis ultimately suggested that ZYX expression was substantially associated with the Rap1 signaling pathway. The human Rap1 gene encodes guanosine triphosphatase (GTPase), which plays a role in cell adhesion and integrin in various cells [26,27]. Rap1 plays an important role in cell adhesion via α5β1 integrin induced by the extracellular matrix molecule fibrinoconnexin and is involved in the regulation of the metastasis mechanism of neck squamous cell carcinoma via the RAP1/RAC1 signaling axis [28]. According to research by Maywald et al., Rap1 controls adhesion proteins in podocytes, which has an impact on the formation and upkeep of the slit septum. Thus, abnormal changes in Rap1 expression may be an important cause of macroproteinuria [29]. Moreover, as a small GTPase, the sequence of Rap1 is highly consistent with that of Ras [30]. Ras and Rap1 work together to initiate and maintain ERK signaling, which is activated in many malignancies and is an important therapeutic target for successful tumor treatment [21,31,32]. We, therefore, speculate that the MEK/ERK (MAPK) signaling pathway may be involved in the mechanism of ZYX in osteosarcoma.

After analyzing the molecular domain, we found that ZYX can interact with Rap1 to affect the downstream MEK/ERK signaling pathway and may ultimately influence the proliferation, migration, and invasion of osteosarcoma. The MEK/ERK (MAPK) signaling pathway is a well-defined pathway in tumor biology, and its aberrant activation is responsible for more than 40% of human tumorigenesis [33,34]. Wang et al. reported that Baicalin, an antioxidant, upregulated DEPP expression and activated its downstream Ras/Raf/MEK/ERK signaling pathway to regulate colon cancer cell senescence [35]. In osteosarcoma, Cyr61 was found to promote the migration and invasion of osteosarcoma by regulating the Raf-1/MEK/ERK signaling pathway [36]. COPS3 interacts with Raf-1 and activates MEK/ERK signaling to regulate osteosarcoma metastasis via autophagy [37]. To identify the regulatory effect of the ZYX/Rap1/MEK/ERK axis on the biological behavior of osteosarcoma, 143B and U2OS cell lines were transfected with Rap1-shRNAs. We discovered that downregulating Rap1 dramatically lessened the inhibitory effect of ZYX on the growth, migration, and invasion of osteosarcoma cells. Hence, we deduced that ZYX suppresses osteosarcoma cell proliferation, migration, and invasion by regulating the Rap1/MEK/ERK signaling pathway.

## 5. Conclusions

In conclusion, our study suggests that ZYX acts as a tumor suppressor gene in osteosarcoma and that its low expression predicts poor prognoses in patients with osteosarcoma. Mechanistically, ZYX interacted with Rap1 to regulate the MEK/ERK signaling pathway, which suppressed osteosarcoma cell proliferation, migration, and invasion. This study, to our knowledge, is the first to describe the relationship between ZYX and the Rap1 signaling pathway, illuminating a novel mechanism underlying osteosarcoma proliferation, migration, and invasion. Our findings add to our understanding of how osteosarcoma progresses and offer a potentially effective treatment target.

## Figures and Tables

**Table 1:** Correlation analyses of ZYX protein expression in relation to clinicopathologic variables of 40 patients with osteosarcoma. * *p <* 0.05; ** *p <* 0.01.

Characteristics	Total	ZYX Expression		*p* Value
		High	Low	
Gender				*p* = 0.7397
Male	24	10	14	
Female	16	5	11	
Age				*p* = 0.5048
≤18 years	28	13	15	
>18 years	12	4	8	
Recurrence				
Yes	21	3	18	*p* = 0.0171 *
No	19	9	10	
Metastasis				
Yes	24	4	20	*p* = 0.0059 **
No	16	10	6	
Clinical stage				
I/II	29	17	12	*p* = 0.0341 *
III/IV	11	2	9	

**Table 2:** Details of the antibodies.

Antibodies	Company	Article Number	Species	Dilutions
ZYX	Proteintech	10330-1-AP	Rabbit	1:1000
Rap1	Servicebio	GB11608-100	Rabbit	1:1000
ERK	Servicebio	GB11560-100	Rabbit	1:1000
p-ERK	Servicebio	GB11004-100	Rabbit	1:1000
MEK	CST	4694S	Mouse	1:1000
p-MEK	CST	9154S	Rabbit	1:1000
GAPDH	Servicebio	GB11002	Rabbit	1:1000

**Table 3:** Primer sequences used for qRT-PCR.

**GAPDH**	F: 5′-GGAAGCTTGTCATCAATGGAAATC-3′
	R: 5′-TGATGACCCTTTTGGCTCCC-3′
**ZYX**	F: 5′-ACTGTGTCCCCGACTACCACAAG-3′
	R: 5′- GACCACTCGCACAGTCTCATCTCG-3′

## Data Availability

All the datasets used and/or analyzed in our study are available.
